# Identification of Dietary Patterns Related to Metabolic Diseases and Their Association with Cardiovascular Disease: From the Korean Genome and Epidemiology Study

**DOI:** 10.3390/nu11102434

**Published:** 2019-10-12

**Authors:** Hye Ah Lee, Hyoin An, Hyesook Park

**Affiliations:** 1 Clinical Trial Center, Mokdong Hospital, Ewha Womans University, Seoul 07985, Korea; 2 Department of Statistics, Ewha Womans University, Seoul 03760, Korea; anhyoin93@gmail.com; 3 Department of Preventive Medicine, College of Medicine, Ewha Womans University, Seoul 07804, Korea; hpark@ewha.ac.kr

**Keywords:** cardiovascular disease, metabolic disease, dietary patterns, cohort study

## Abstract

Using data from the community-based cohort of the Korean Genome and Epidemiology Study (KoGES), we evaluated the dietary patterns (DPs) related to metabolic diseases and their associations with the incidence of non-fatal cardiovascular disease (CVD). After excluding those with a history of CVD or cancer, we analyzed the data of 8352 subjects aged 40–69 years. Based on their daily intake of 26 food groups at baseline, the DPs of the subjects with metabolic diseases (*n* = 1679, 20.1%) were analyzed using principal component analysis. Due to regional differences in the effect of DPs on CVD, we performed analyses stratified by region. The association between DPs and the incidence of non-fatal CVD was evaluated by calculating the hazard ratio (HR) and 95% confidence interval (CI) using the Cox proportional hazards model. During the 12-year follow-up, the incidence of non-fatal CVD was 5.4 per 1000 person-years (*n* = 431). An animal-based DP made the greatest contribution to the total variance and was characterized by a high intake of pork, beef, chicken, fish, and shellfish. The effect of DP on CVD differed by region (industrial/rural regions, *p* < 0.05) and was dominant in industrial regions, irrespective of metabolic disease status. In industrial regions, subjects in the top quintile of DP had a 0.42-fold (95% CI = 0.24–0.74) lower risk of incident CVD than those in the bottom quintile, even after adjusting for various covariates. In addition, the risk of CVD was high in individuals with a history of metabolic disease in both regions (HR = 1.74, 95% CI = 1.24–2.43 in industrial regions; HR = 1.88, 95% CI = 1.42–2.48 in rural regions). DP and a history of metabolic diseases, but not their interaction, were independently associated with incident CVD. In our study, an animal-based DP related to metabolic disease was independently associated with incident CVD, and this effect was noticeable only in industrial regions.

## 1. Introduction

Metabolic risk factors such as elevated blood pressure and abnormal levels of glucose or lipids are risk factors for cardiovascular disease (CVD) [1]. Over the past two decades, the CVD mortality rate has declined markedly worldwide [2], but ischemic heart disease and stroke are still leading causes of death and combined accounted for 18.8% of all deaths in South Korea in 2017 [3]. Accordingly, CVD is a major public health issue in South Korea, where hypertension (HTN) and diabetes prevention and management programs are being implemented in community health centers. The primary-care guidelines for HTN and diabetes recommend reducing salt intake, eating vegetable-based meals, increasing dietary fiber intake (whole grains), and weight control [4,5]. Such guidelines typically focus on a single food or nutrient.

Several studies have aimed to identify dietary patterns (DPs) related to CVD, because DPs reflect the actual dietary behavior of the population and provide comprehensive information on dietary behaviors [6]. Two large prospective cohort studies in the United States (US) found that prudent and Western DPs were negatively and positively associated with the risk of coronary heart disease (CHD), respectively [7,8]. The Caerphilly Prospective Study reported that a DP characterized by higher intake of white bread, butter, and sugar-sweetened beverages, and lower intake of wholegrain bread was related to a greater risk of CVD and stroke in middle-aged British males [6]. Although the evidence implies that several DPs are related to circulatory diseases, the traditional DPs of Koreans differ from those of Western countries. In addition, a meta-analysis revealed that the effect of DP on health varies from country to country [9].

Persons with metabolic diseases such as HTN, dyslipidemia, and diabetes mellitus (DM) have a higher risk of CVD compared to healthy individuals [1]. Taking account of the increasing prevalence of metabolic diseases [10], research is needed to identify modifiable factors that can prevent CVD in subjects with a metabolic disease. In addition, most previous studies considered metabolic diseases only as confounding variables in evaluating the relationship between DPs and incident CVD.

Therefore, using population-based cohort data from South Korea, we identified the DPs of subjects with metabolic diseases using principal component analysis (PCA). DP scores were calculated based on the results of PCA to assess whether selected DPs differed significantly from those without metabolic diseases. Then, we assessed the association between selected DPs and incident CVD, as well as assessing interaction effects with various potential risk factors.

## 2. Subjects and Methods

### 2.1. Subjects

This study used data from the community-based cohort of the Korean Genome and Epidemiology Study (KoGES). All participants in the KoGES study provided informed consent for participation in the study [11]. The KoGES data are available on request from the National Research Institute of Health [11]. Detailed information about the KoGES data has been reported [11]. Briefly, the community-based cohort of the KoGES was recruited from among 40–69 years old residents of the Ansung (rural region, *n* = 5018, response rate = 69.6%) and Ansan (industrial region, *n* = 5012, response rate = 45.7%) communities in Gyeonggi Province. The subjects completed the baseline survey in 2001 and 2002. The cohort was designed to evaluate risk factors for non-communicable diseases in Koreans. Follow-up is conducted at two-year intervals and is currently ongoing. The follow-up consists of questionnaires, anthropometric measurements, blood sampling, urine tests, and biomarker assessments, performed by trained technicians and interviewers. Data from the sixth follow-up survey, conducted in 2013–2014, are available (follow-up rate = 62.8%).

In this study, subjects who met any of the following criteria were excluded: missing data for the baseline dietary survey (*n* = 326); daily reported caloric intake of <500 or >5000 kcal (*n* = 84); or history of cancer, myocardial infarction, stroke, coronary artery disease, or congestive heart failure (*n* = 1268). Finally, 8352 subjects (3987 males and 4365 females) were included in this study. Among them, 1679 subjects (20.1%) had a history of HTN (*n* = 1211, 14.5%), dyslipidemia (*n* = 196, 2.3%), or DM (*n* = 534, 6.4%), and 250 subjects reported having two or more of these diseases (Supplementary Figure S1). The median disease duration was three years (interquartile range = 1–8 years). The subjects excluded from this study were slightly older and more likely to have a low socioeconomic status than those who were included. However, there was no difference in body mass index (BMI) or the proportion of current smokers (data not shown). The study protocol was approved by the Institutional Review Board (IRB) of Ewha Womans University Hospital (IRB no. EUMC 2017-06-041).

### 2.2. Food Intake Data and DP Assessment

A baseline dietary survey was conducted by dieticians using a validated, dish-based semi-quantitative food-frequency questionnaire (FFQ) [12]. The FFQ covers 103 food items and provides information on average food intake during the past year using a nine-point frequency scale (<1 time/month or never, 1 time/month, 2–3 times/month, 1–2 times/week, 3–4 times/week, 5–6 times/week, 1 time/day, 2 times/day, and 3 times/day) and a three-point scale for amount consumed (less than average, average, and more than average). The KoGES provides daily intake data (grams per day) for the 103 food items. Similar food items were pooled into 26 food groups, as reported previously [13]. Energy-adjusted intakes were calculated using a residual method and used in the further analysis.

Using principal component analysis (PCA), we identified the DPs of subjects with metabolic diseases (*n* = 1679) at baseline according to the energy-adjusted daily intake of 26 food groups. Along with eigenvalues and scree plots, the first three factors were determined by considering the subgroup analysis results according to the type of metabolic disease. The DPs identified were similar between males and females, so the analysis was not stratified by sex. To simplify interpretation of the results, the factors were rotated by an orthogonal method. Food groups with a factor loading value of ≥0.2 were considered major contributors to a DP. Therefore, the first three DPs, based on this criterion, were named the animal-based DP, healthy DP, and Western DP.

### 2.3. Outcomes

Incident non-fatal CVD was assessed by questionnaire for the diseases diagnosed by physicians. Myocardial infarction, stroke, coronary artery disease, and congestive heart failure were considered as CVD-related diseases. Follow-up began upon entry into the study and ended on the date of physician-diagnosed CVD or the last follow-up, whichever was sooner. Those who died during follow-up before the CVD incident were treated as censored. Regarding the validity of the data, 93% agreement between self-reported disease diagnosis and diagnosis made by reference to medical records was seen [14].

### 2.4. Covariates

HTN, dyslipidemia, and DM were considered metabolic diseases. Metabolic disease history was determined based on the answer to a question in the baseline survey regarding whether a disease was physician-diagnosed. The numbers and types of metabolic diseases were included in the multivariate model. Alternatively, as a metabolic index, baseline systolic blood pressure, high-density lipoprotein cholesterol level, and fasting plasma glucose level were included in a multivariate model and analyzed.

As demographic factors, age, sex, region (industrial/rural), and educational level at baseline were analyzed. Among available data, we also included current smoking [1,8], alcohol intake (no alcohol, <15 g/day, 15–24.9 g/day, ≥25 g/day) [8], quartile of physical activity [1,8,15], parental history of CVD (yes or no) [8], and BMI (continuous) [8] as CVD-related risk factors. Based on the International Physical Activity Questionnaire [16], physical activity over the past year was measured as metabolic equivalent of task (MET)-hours per week, which takes into account both the intensity and duration of physical activity.

### 2.5. Statistical Analysis

Descriptive results are summarized as means with the standard deviation for numeric variables and frequency with percentage for categorical variables. Baseline characteristics were compared according to history of metabolic disease by Student’s *t*-test and the chi-squared test.

Based on the results of the PCA, to assess the effects of DPs, the baseline DP score was calculated including the subjects without metabolic disease. In univariate analyses, we assessed the associations between the DPs and non-fatal CVD; only an animal-based DP showed a significant association. Thus, the animal-based DP was included in all subsequent analyses. As covariates, we included age, sex, region, educational level, current smoking, alcohol intake, quartile of physical activity, parental history of CVD, BMI, and number of metabolic diseases at baseline. In the interaction evaluation, the effect of DP on the incidence of CVD differed by region (*p* < 0.05); thus, we performed further analyses stratified by region. There was no significant interaction effect except for the region.

The incidence of non-fatal CVD was estimated per 1000 person-years. We estimated the effect of DP on the incidence of CVD by calculating the hazard ratio (HR) and 95% confidence interval (CI) using the Cox proportional hazards model. The dose-response relationship between DP and incident CVD was also evaluated. The estimated effect was calculated by adjusting the covariates mentioned above. The type of metabolic disease or metabolic index (systolic blood pressure, high-density lipoprotein cholesterol level, and fasting glucose level) were also evaluated. Additionally, disease duration from the first diagnosis of metabolic disease was included as a covariate. The assumptions of the Cox proportional hazards model were satisfied, as assessed by the Schoenfeld residuals method. In sub-analyses, we evaluated the effect of DP on myocardial infarction, stroke, and coronary artery disease, as well according to current smoking, obesity, and age at baseline (cutoff of <60 years). Statistical analyses were conducted using SAS software (version 9.4; SAS Institute, Cary, NC, USA). Statistical significance was determined as a value of *p* < 0.05 in a two-tailed test.

## 3. Results

At baseline, the average age of the subjects was 52.0 years and 47.7% were males. Half of the subjects lived in rural regions. Compared to those without a metabolic disease, individuals with a metabolic disease at baseline were more likely to be older and obese, live in a rural region, and have a low educational level. However, there was no difference in sex, physical activity, parental history of CVD, total energy, or protein intake (Table 1). For food group intake, the average intake of sugar, pork, chicken, and dairy products was lower in individuals with versus those without a metabolic disease; the reverse association was found for fish intake (Supplementary Table S1).

**Figure 1:**
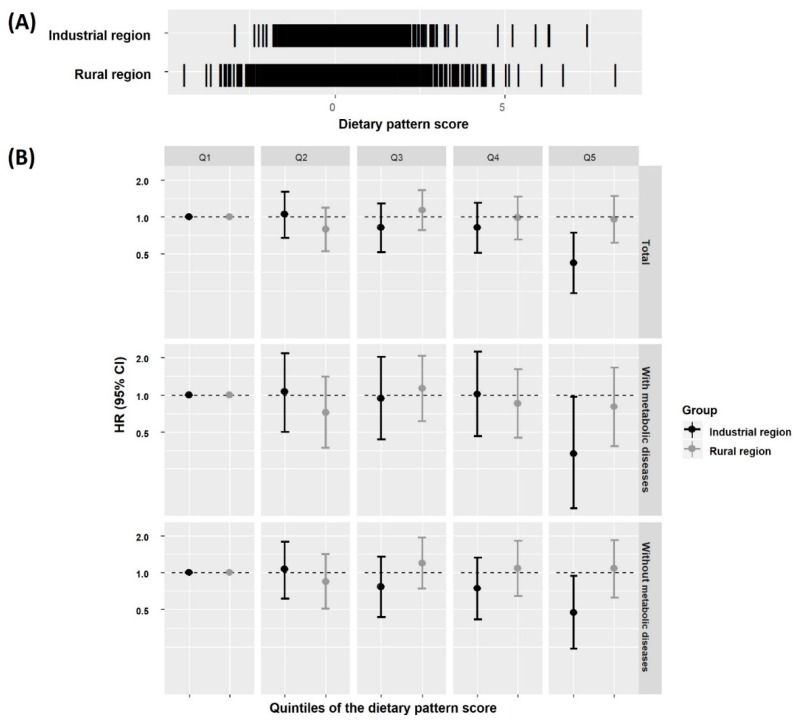
Regional distribution of animal-based dietary pattern (DP) scores (**A**) and effect of animal-based DP score quintile (Q) on incident non-fatal cardiovascular diease (CVD) according to region and history of metabolic disease at baseline (**B**). Hazard ratios (HRs) with 95% confidence interval (CI) were estimated with adjustment for age, sex, educational level, current smoking, alcohol intake, physical activity quartile, parental history of CVD, and body mass index (BMI). The number of metabolic diseases at baseline was considered in subjects with a metabolic disease.

Table 2 shows the factor loading values according to DP of subjects with a metabolic disease at baseline (*n* = 1679). The animal-based DP explained the largest proportion (38.4%) of the total variance and was characterized by high intake of pork, beef, chicken, fish, and shellfish. The second DP was predominantly fruit, vegetables, and seaweeds, and therefore was named the healthy DP. The third DP (Western DP) was characterized by a high intake of sugar, bread, coffee, and noodles. The mean DP scores varied according to baseline metabolic status (Table 3). The mean animal-based and Western DP scores were slightly higher in subjects without metabolic disease than in those with a metabolic disease, but the healthy DP showed the opposite tendency. These results were similar to the pattern of results according to the absence or presence of hypertension. Of the DPs, only the animal-based DP score differed significantly according to incident CVD (Table 3). Also, the animal-based DP score distribution varied by region (Figure 1), as did the effect of DP on incident CVD (*p* < 0.05).

During the 12-year follow-up, 431 subjects were diagnosed with a CVD by a physician (incidence = 5.4 per 1000 person-years). The incidence was slightly higher in rural regions (*n* = 256, 6.5 per 1000 person-years) than in industrial regions (*n* = 175, 4.3 per 1000 person-years). Compared to rural regions, the average age, physical activity level, and metabolic disease rate were lower, and the educational level and the obesity rate were higher, in industrial regions (Supplementary Table S2). However, in both regions, the subjects with a high animal-based DP score were predominantly male and tended to be younger and more educated (Supplementary Tables S3 and S4).

The effect of animal-based DP on the incidence of non-fatal CVD is shown in Table 4. Compared to those in the bottom quintile, those in the top quintile had a 0.77-fold (95% CI = 0.52–1.14) and 0.42-fold (95% CI = 0.24–0.72) lower risk of incident CVD in rural and industrial regions, respectively. The addition of covariates to the model resulted in a HR of DP in rural regions of almost 1.0. In contrast, the effect of DP on CVD in industrial regions remained significant after adjusting for various covariates (Table 4), and for the duration of metabolic disease (data not shown), with a significant dose-response relationship. The results were similar irrespective of the presence of a metabolic disease at baseline (Figure 1). In regard to the type of CVD, the effect of DP was significant in coronary artery disease and stroke, but not in myocardial infarction. The effect of the animal-based DP on CVD was also significant in subjects with a BMI ≥25.0 kg/m^2^ and those less than 60 years of age. Those with top quintile of DP in current smokers had a lower risk of CVD, albeit with only borderline significance (*p* = 0.09) (Supplementary Table S5).

**Figure 2:**
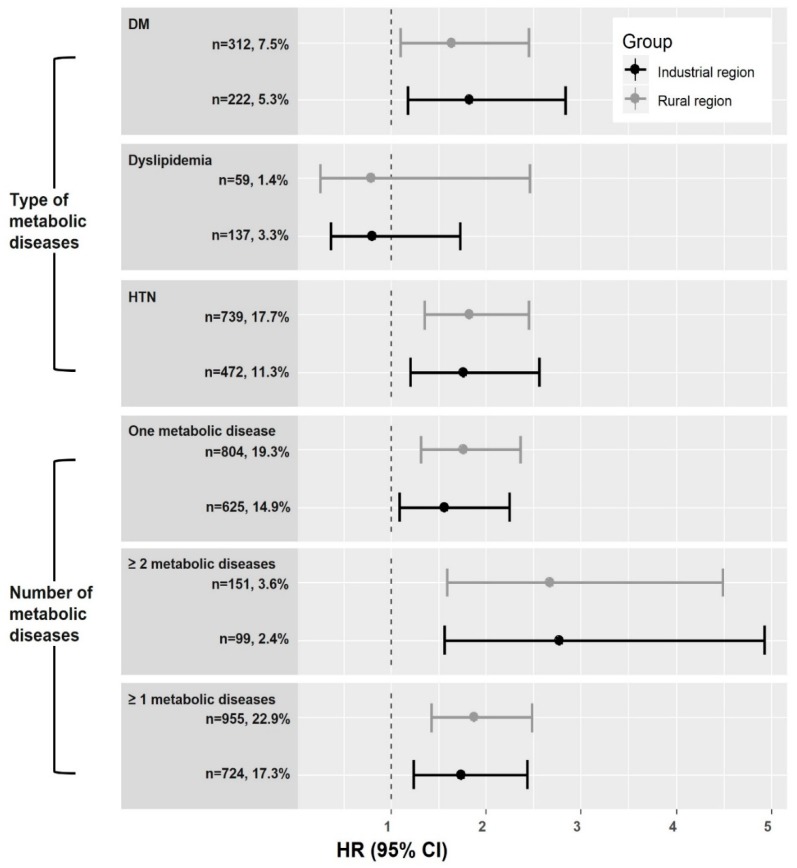
Effect of metabolic status on incident non-fatal CVD according to region of residence. DM, diabetes mellitus; HTN, hypertension; CVD, cardiovascular disease. The hazard ratios (HRs) with 95% confidence interval (CI) were estimated after adjustment for age, sex, educational level, current smoking, alcohol intake, physical activity quartile, parental history of CVD, body mass index, and animal-based dietary pattern (DP) score quintile.

As expected, subjects with a metabolic disease at baseline had a higher risk of incident CVD, irrespective of region. Subjects with one of HTN, dyslipidemia, or DM had a 1.6- to 1.8-fold increased risk of CVD, while those with two or more of these metabolic diseases at baseline had a 2.7- to 2.8-fold increased risk of CVD compared to those without. Diabetes and HTN were independently associated with incident CVD, irrespective of region (Figure 2). Systolic blood pressure and fasting plasma glucose level, but not high-density lipoprotein-cholesterol level, were independently associated with incident CVD (data not shown). An animal-based DP and a history of metabolic disease were independently associated with incident CVD, with no interaction.

## 4. Discussion

Using longitudinal cohort data, we evaluated the DPs of middle-aged Koreans with a history of metabolic disease. An animal-based DP was inversely and dose-dependently associated with non-fatal CVD in industrial regions but not in rural regions. This effect was significant irrespective of the presence of a metabolic disease at baseline. The effect of an animal-based DP on CVD remained significant in subjects with obesity and those less than 60 years of age. As expected, subjects with a metabolic disease at baseline were at greater risk of incident CVD, but this relationship was not modulated by the level of an animal-based DP.

Individuals with metabolic diseases are at higher risk of CVD than disease-free individuals [1]. However, little research has focused on the dietary requirements of such people. Most previous studies have focused on healthy people or been performed without a clear distinction of disease history. Several studies investigated DPs in subjects with type 2 diabetes, but most of these studies were cross-sectional or lacked a control group [17,18]. In addition, prior cohort studies analyzed only metabolic diseases at baseline as covariates [8,19]. Here, we evaluated the DPs of subjects with a history of metabolic disease who may be at high risk of CVD. The identified DPs of subjects with a history of metabolic disease were similar to those without a history of metabolic disease (data not shown). It seems that the major DPs with considerable explanatory power are similar in subgroups of the population. Similarly, the extant studies on DPs differ in methodology and the region in which they were conducted, but a healthy DP and Western DP have been reported in many countries [7,8,9]. A cross-sectional study in South Korea found three DPs in metabolic disease populations, similar to our results [20]. In the present study, the mean healthy DP score was higher in subjects with a metabolic disease than in those without (Table 3), which likely reflects the fact that the former follow a healthier diet to manage their disease. However, only an animal-based DP was significantly related to incident CVD. In line with our study, a previous study based on the same data also found that moderate intake of unprocessed red meat or poultry was associated with a reduced risk of CVD [21].

The effect of consumption of animal-derived food on CVD is controversial. A systematic review reported that the effect of unprocessed red meat on CVD mortality was significant in the US population, but not in European or Asian populations [22]. A pooled analysis of eight Asian prospective cohort studies showed that red meat intake was inversely associated with all-cause mortality in both sexes, and with CVD mortality in males [23]. This may be due to differences in meat intake by geographical region. Although the gap in meat intake between Western and Asian countries has decreased, the average meat intake of Asian countries is lower than that of Western countries (consumption of beef, veal, pork, and poultry in 2017: 97.9 kg per capita in the US, 67.2 kg in the European Union, and 57.3 kg in South Korea) [23,24]. Use of different food preparation and cooking methods may also be a contributing factor [21].

In this study, subjects in the top quintile of the animal-based DP were more educated and had a higher income (Supplementary Tables S3 and S4). A recent systematic study of data from Europe, the US, and Asia revealed that lower educational level and lower income were associated with an increased risk of CHD, stroke, and CVD [25]. Socioeconomic factors are related to living environment, lifestyle, and health literacy [25]. Socioeconomic factors are also closely linked to food choice, resulting in differences in diet quality [26]. Therefore, in this study, the socioeconomic factors seemed to contribute significantly to the effects of DPs on incident CVD. Also, an animal-based DP was most closely correlated with protein intake (partial *r* = 0.84, *p* < 0.0001), followed by niacin (vitamin B3) and vitamin B1 (partial *r* = 0.77 and 0.62, respectively, *p* < 0.0001). Although the effects of niacin and protein on CVD are controversial [27,28], nicotinic acid reportedly improves the blood lipid profile [28] and protein intake improves blood pressure [29]. A previous study based on the same data reported that a low intake of meat was independently associated with the incidence of HTN [30]. In this study, the beneficial effect of an animal-based DP may have been mediated in part by these nutrients.

The regional difference in the effect of an animal-based DP on the risk of CVD seen in this study may have been due to demographic factors, cultural differences, and/or food accessibility. To control for regional differences in age distribution, which is a major determinant of the risk of CVD, we excluded persons >60 years of age from sub-group analyses, but the results were unaffected. Protein intake was generally adequate in industrial regions, according to the recommended level, whereas one in four subjects in rural regions had insufficient protein intake (95.7% vs. 75.2%, *p* < 0.0001). There seems to have been a difference in the quality of diet due to differences in socioeconomic status among the study regions. However, since our data included only two communities, it is difficult to discuss regional disparities in health risks. Further studies are needed to assess the above-mentioned effects in other regions of South Korea.

This study had several limitations. First, the data were derived from only two communities and therefore are not representative of the entire population of South Korea, which hampers generalization of the results. Second, for reasons of data accessibility, we included only non-fatal CVD cases. Thus, further studies should also analyze the data of subjects who died due to CVD. Third, the dietary survey was conducted using a validated FFQ, but there may have been measurement errors in the estimation of daily intake. Finally, residual confounding by unmeasured factors, such as changes in diet during the follow-up period and bias due to loss to follow-up, might have influenced the results.

This study also had strengths. The effect of DPs on CVD were evaluated including subjects without a metabolic disease as controls, as was the likelihood of interactions with prevalent diseases. We assessed regional differences in the effects of DPs on CVD and found that there were significant interaction effects. Furthermore, the data were from a cohort study with long-term follow-up, and are therefore instructive regarding causality. Further studies are required to validate our findings, but our study provides evidence for Asian countries for which similar studies are currently lacking.

## 5. Conclusions

In summary, we evaluated the DPs of subjects with a history of metabolic disease and assessed their associations with the incidence of non-fatal CVD. Irrespective of the presence of metabolic disease at baseline, an animal-based DP was independently associated with a reduced risk of CVD in industrial regions, but not in rural regions. Further studies are required to validate our findings.

## Figures and Tables

**Table 1:** Baseline characteristics according to metabolic disease status.

Characteristic	Total (*n* = 8352)	Subjects with Metabolic Diseases (*n* = 1679, 20.1%)	Subjects without Metabolic Diseases (*n* = 6673, 79.9%)	*p*
Age (years)	52.02 ± 8.84	55.68 ± 8.54	51.10 ± 8.67	<0.0001
Male	3987 (47.74)	774 (46.10)	3213 (48.15)	0.14
Rural region	4169 (49.92)	955 (56.88)	3214 (48.16)	<0.0001
Education level				
Under high school	4613 (55.54)	1012 (60.74)	3601 (54.24)	<0.0001
Graduated high school	2555 (30.76)	414 (24.85)	2141 (32.25)	
Some college or higher	1137 (13.69)	240 (14.41)	897 (13.51)	
Monthly income (KRW), %				
<1,000,000	2821 (34.26)	699 (42.13)	2122 (32.27)	<0.0001
1,000,000 ≤ 1,999,999	2428 (29.48)	461 (27.79)	1967 (29.91)	
≥2,000,000	2986 (36.26)	499 (30.08)	2487 (37.82)	
BMI (kg/m^2^)	24.59 ± 3.13	25.68 ± 3.13	24.31 ± 3.08	<0.0001
Normal (<23 kg/m^2^)	2594 (31.07)	325 (19.39)	2269 (34.01)	<0.0001
Overweight (23–24.9 kg/m^2^)	2192 (26.26)	387 (23.09)	1805 (27.05)	
Obese (≥25 kg/m^2^)	3562 (42.67)	964 (57.52)	2598 (38.94)	
Current smoking	2102 (25.38)	354 (21.24)	1748 (26.42)	<0.0001
Alcohol intake (g/day)				
Non-intake	4321 (52.99)	960 (58.32)	3361 (51.64)	<0.0001
<15.0 g/day	2258 (27.69)	368 (22.36)	1890 (29.04)	
15.0–24.9g/day	567 (6.95)	118 (7.17)	449 (6.90)	
≥25.0 g/day	1009 (12.37)	200 (12.15)	809 (12.43)	
Physical activity (MET-hours/week)				
Q1 (<25th)	1885 (22.57)	381 (22.69)	1504 (22.54)	0.94
Q2 (25–49th)	2289 (27.41)	466 (27.75)	1823 (27.32)	
Q3 (50–74th)	2089 (25.01)	410 (24.42)	1679 (25.16)	
Q4 (≥75th)	2089 (25.01)	422 (25.13)	1667 (24.98)	
Parental history of CVD	306 (3.66)	58 (3.45)	248 (3.72)	0.66
Total energy (kcal)	1943.78 ± 622.2	1924.1 ± 643.3	1948.7 ± 616.7	0.16
Carbohydrate (g/day)	342.89 ± 36.34	346.6 ± 36.62	342.0 ± 36.21	<0.0001
Protein (g/day)	65.96 ± 12.32	65.84 ± 13.19	66.00 ± 12.09	0.66
Fat (g/day)	32.11 ± 12.18	30.45 ± 12.14	32.52 ± 12.16	<0.0001

KRW, Korean Won; BMI, body mass index; MET, metabolic equivalent of task; CVD, cardiovascular disease; Q, quartile.

**Table 2:** The dietary patters (DPs) of subjects with a metabolic disease: results of principal component analysis.

Food Group	Subjects with Metabolic Diseases (*n* = 1679)		
	DP 1	DP 2	DP 3
Pork	0.58		
Shellfish	0.46	0.24	
Beef	0.46		
Fish	0.45	0.34	
Chicken	0.43		
Mushrooms	0.39	0.34	
Other meat	0.38		
Other drinks	0.21		
Fruit		0.54	
Vegetables	0.20	0.51	
Seaweeds		0.38	
Potatoes		0.24	
Soybean		0.24	
Eggs		0.21	
Milk			
Sugar			0.54
Bread			0.44
Coffee			0.34
Noodles		−0.24	0.32
Carbonated drink			0.26
Dairy products		0.22	0.24
Processed meat	0.22		0.23
Oil and fat			0.22
Nuts and seeds			
Kimchi			
Rice	−0.48	−0.54	−0.59
Variance explained (%)	38.4	17.6	12.7

Factor loading values are shown; values < │0.2│ are not presented.

**Table 3:** DP score according to metabolic disease status at baseline or incident CVD.

	DP1 Score		DP2 Score		DP3 Score	
	Means	SD	Means	SD	Means	SD
At baseline						
with metabolic diseases (*n* = 1679)	0.00	0.85	0.00	0.90	0.00	0.90
without metabolic diseases (*n* = 6673)	0.10	0.83	−0.06	0.85	0.16	0.93
*p*	<0.0001		0.01		<0.0001	
with DM (*n* = 534)	0.05	0.93	0.04	1.03	−0.01	0.96
without DM (*n* = 7818)	0.08	0.82	−0.06	0.84	0.14	0.92
*p*	0.40		0.03		<0.001	
with dyslipidemia (*n* = 196)	0.16	0.77	−0.07	0.90	0.24	0.85
without dyslipidemia (*n* = 8156)	0.08	0.83	−0.05	0.86	0.12	0.93
*p*	0.17		0.76		0.09	
with HTN (*n* = 1211)	−0.03	0.85	0.02	0.87	−0.03	0.90
without HTN (*n* = 7141)	0.10	0.83	−0.06	0.85	0.15	0.93
*p*	<0.0001		<0.01		<0.0001	
During the follow-up period						
with incident CVD (*n* = 431)	−0.06	0.85	−0.09	0.80	0.04	0.88
without incident CVD (*n* = 7921)	0.09	0.83	−0.05	0.86	0.13	0.93
*p*	<0.001		0.24		0.06	

DM, diabetes mellitus; HTN, hypertension; CVD, cardiovascular disease; DP, dietary pattern; SD, standard deviation.

**Table 4:** Association between animal-based DP score quintile at baseline and incident CVD.

	Model	Quintiles (Q) of Animal-Based DP Score					*p* for Trend
		Q1	Q2	Q3	Q4	Q5	
Rural region	Incident case/person-years	58/7961.9	46/7697.4	58/7745.1	50/7936.4	44/7767.6	
	Univariate ^1^	Ref	0.79 (0.53–1.17)	1.01 (0.7–1.46)	0.85 (0.58–1.25)	0.77 (0.52–1.14)	0.31
	Model 1 ^1^	Ref	0.78 (0.53–1.16)	1.08 (0.75–1.56)	1.01 (0.69–1.48)	1.00 (0.67–1.50)	0.63
	Model 2 ^1^	Ref	0.79 (0.53–1.18)	1.13 (0.78–1.65)	0.98 (0.66–1.46)	0.96 (0.62–1.47)	0.79
	Model 3 ^1^	Ref	0.79 (0.53–1.19)	1.14 (0.79–1.66)	0.99 (0.67–1.46)	0.96 (0.62–1.47)	0.79
Industrial region	Incident case/person-years	42/8128.4	46/8160.4	36/8364.3	33/8199.7	18/8223.2	
	Univariate ^1^	Ref	1.07 (0.70–1.62)	0.8 (0.51–1.25)	0.76 (0.48–1.20)	0.42 (0.24–0.72)	<0.001
	Model 1 ^1^	Ref	1.05 (0.69–1.59)	0.78 (0.50–1.23)	0.81 (0.51–1.28)	0.43 (0.24–0.75)	0.002
	Model 2 ^1^	Ref	1.05 (0.68–1.60)	0.81 (0.52–1.28)	0.82 (0.51–1.30)	0.42 (0.24–0.74)	0.002
	Model 3 ^1^	Ref	1.03 (0.67–1.58)	0.79 (0.50–1.25)	0.82 (0.51–1.30)	0.41 (0.23–0.72)	0.002

DP, dietary pattern; CVD, cardiovascular disease ^1^ Values are hazard ratios with 95% confidence intervals. Model 1: Adjusted for age and sex. Model 2: Adjusted as in model 1 plus educational level, smoking status, alcohol intake, quartile of physical activity, parental history of cardiovascular disease, body mass index, and number of metabolic diseases at baseline. Model 3: Adjusted as in model 1 plus educational level, smoking status, alcohol intake, quartile of physical activity, parental history of cardiovascular disease, body mass index, history of diabetes mellitus, history of dyslipidemia, and history of hypertension.

## Supplementary Materials

The following are available online at https://www.mdpi.com/2072-6643/11/10/2434/s1, Figure S1: title, Table S1: title, Video S1: title.
