# Supplementary material for: Identification of Dietary Patterns Related to Metabolic Diseases and Their Association with Cardiovascular Disease: From the Korean Genome and Epidemiology Study
Source: Nutrients. 2019 Oct 12;11(10):2434. doi: 10.3390/nu11102434 (PMC6835220; doi:10.3390/nu11102434)
Supplement: Supplementary file 1 [file nutrients-11-02434-s001.pdf]

Supplemental Figure S1. Sample size and metabolic disease history of at baseline

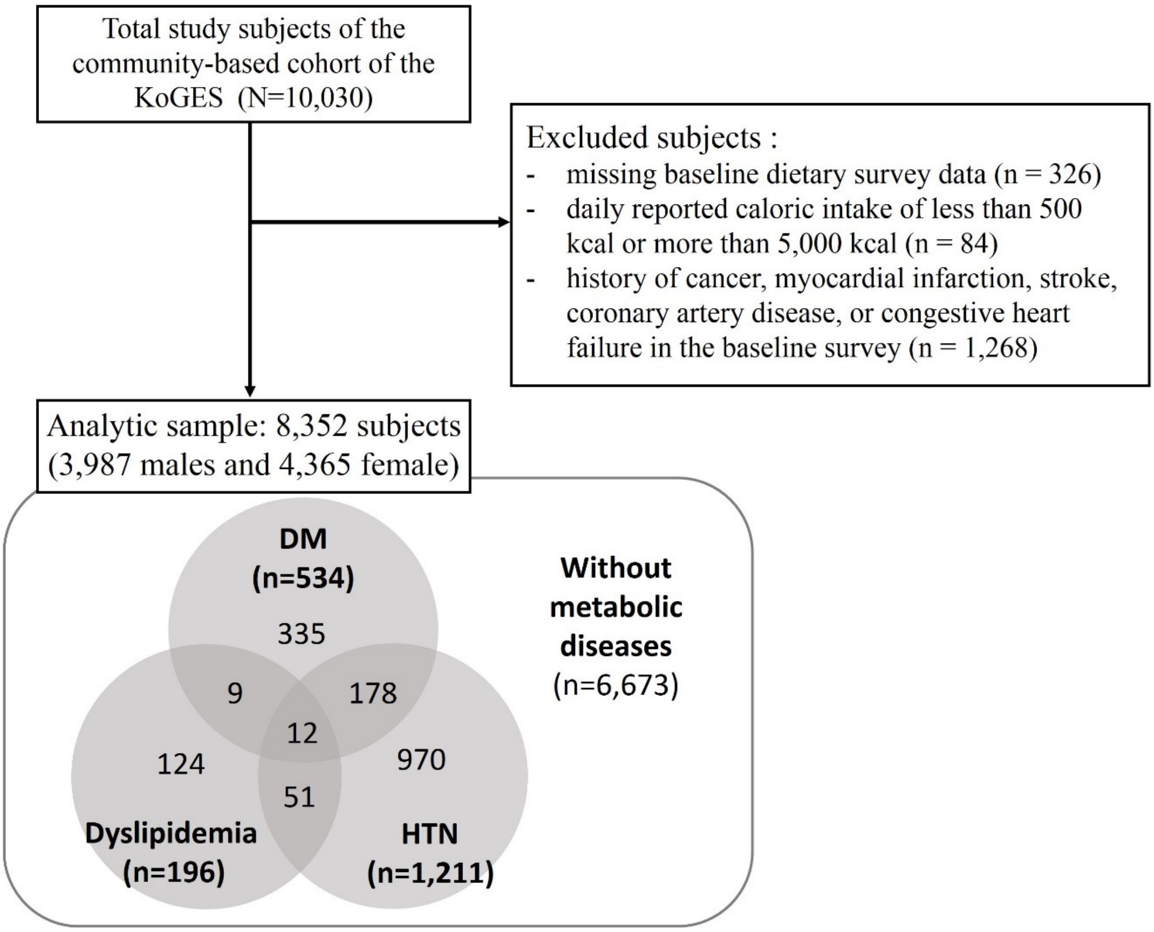

Data are numbers of subjects.

Supplemental Table S1. Food intake according to history of metabolic disease at baseline.

Foods	Subjects with metabolic diseases (n = 1,679, 20.1%)			Subjects without metabolic diseases (n = 6,673, 79.9%)			*P*
	LSmeans	95 % CI		LSmeans	95 % CI		
Total energy (kcal)	1950.13	1920.27	1980.00	1942.31	1927.53	1957.10	0.65
Rice	681.10	672.45	689.74	675.57	671.29	679.85	0.26
Noodles	74.28	70.67	77.89	76.06	74.27	77.84	0.39
Bread	11.85	10.79	12.92	12.70	12.17	13.23	0.16
Sugar	14.47	13.59	15.34	16.41	15.97	16.84	<0.001
Oil and fat	0.06	0.04	0.08	0.07	0.06	0.08	0.55
Potatoes	22.73	21.38	24.08	22.60	21.93	23.27	0.87
Soybean	46.99	44.81	49.16	40.82	39.74	41.90	<0.0001
Nuts and seeds	0.68	0.56	0.80	0.80	0.74	0.86	0.08
Kimchi	203.31	196.58	210.04	204.25	200.92	207.58	0.81
Vegetables	130.60	125.40	135.80	125.76	123.19	128.33	0.10
Mushrooms	8.14	7.60	8.69	7.62	7.35	7.89	0.09
Fruit	258.85	245.61	272.09	266.16	259.61	272.72	0.34
Processed meat	1.13	0.96	1.30	1.10	1.01	1.18	0.72
Pork	32.73	31.00	34.46	35.92	35.06	36.77	<0.01
Beef	9.32	8.68	9.97	8.85	8.54	9.17	0.20
Chicken	6.84	6.35	7.33	7.63	7.39	7.87	0.01
Other meat	3.04	2.69	3.40	2.96	2.78	3.13	0.67
Eggs	12.10	11.34	12.87	12.50	12.12	12.88	0.37
Fish	29.43	28.07	30.79	26.82	26.14	27.49	<0.001
Shellfish	13.81	13.01	14.60	14.15	13.76	14.54	0.45
Seaweeds	1.93	1.84	2.02	1.89	1.85	1.94	0.41
Milk	73.95	68.75	79.15	70.63	68.06	73.21	0.27
Dairy products	34.04	31.30	36.78	37.78	36.42	39.13	0.02
Carbonated drink	22.60	20.05	25.14	24.24	22.99	25.50	0.26
Coffee	2.87	2.71	3.03	3.02	2.94	3.10	0.11
Other drinks	56.40	52.39	60.40	48.11	46.13	50.10	<0.001

LSmeans, least-squares means; CI, confidence interval
Food intake was calculated using a residual method to control for total energy intake.
LSmeans with 95% CI were obtained from the generalized linear model after adjusting for age, sex, and region of residence.

Supplemental Table S2. Baseline characteristics of the subjects according to region of residence

**Characteristics**	**Rural region (n= 4,169, 49.9%)**	**Industrial region (n=4,183, 50.1%)**	***P***
Age (years)	55.38±8.74	48.67±7.57	<0.0001
Male	1830 (43.9)	2157 (51.57)	<0.0001
Education level			
Under high school	3144 (76.2)	1469 (35.15)	<0.0001
Graduated high school	738 (17.89)	1817 (43.48)	
Some college or higher	244 (5.91)	893 (21.37)	
Monthly income (KRW), %			
< 1,000,000	2293 (56.26)	528 (12.70)	<0.0001
1,000,000 ≤ 1,999,999	1167 (28.63)	1261 (30.32)	
≥ 2,000,000	616 (15.11)	2370 (56.98)	
BMI (kg/m^2)	24.46±3.30	24.71±2.95	<0.001
Normal (< 23 kg/m^2)	1408 (33.8)	1186 (28.36)	<0.0001
Overweight (23–24.9 kg/m^2)	1033 (24.8)	1159 (27.71)	
Obese (≥ 25 kg/m^2)	1725 (41.41)	1837 (43.93)	
Current smoking	1105 (26.88)	997 (23.9)	<0.01
Alcohol intake (g/day)			
Non-intake	2334 (58.22)	1987 (47.93)	<0.0001
< 15.0 g/day	1023 (25.52)	1235 (29.79)	
15.0–24.9g/day	226 (5.64)	341 (8.22)	
≥ 25.0 g/day	426 (10.63)	583 (14.06)	
Physical Activity[1] (MET-hours/week)			
Q1 (< 25th)	867 (20.8)	1018 (24.34)	<0.0001
Q2 (25–49th)	602 (14.44)	1687 (40.33)	
Q3 (50–74th)	832 (19.96)	1257 (30.05)	
Q4 (≥ 75th)	1868 (44.81)	221 (5.28)	
Parental history of CVD	130 (3.12)	176 (4.21)	<0.01
History of DM	312 (7.48)	222 (5.31)	<0.0001
History of dyslipidemia	59 (1.42)	137 (3.28)	<0.0001
History of HTN	739 (17.73)	472 (11.28)	<0.0001
Number of metabolic diseases			
0	3214 (77.09)	3459 (82.69)	<0.0001
1	804 (19.29)	625 (14.94)	
≥ 2	151 (3.62)	99 (2.37)	
Total energy (kcal)	1969.8±731.5	1917.8±488.3	0.0001
Carbohydrate (g/day)	352.8±40.39	333.0±28.52	<0.0001
Protein (g/day)	63.08±13.63	68.84±10.07	<0.0001
Fat (g/day)	28.95±13.67	35.26±9.50	<0.0001

DM, diabetes mellitus; HTN, hypertension; BMI, body mass index; MET, metabolic equivalent of task; KRW, Korean Won; CVD, cardiovascular disease

Supplemental Table S3. Baseline characteristics of the subjects in the rural region according to animal-based DP score quintile

**Characteristic**	Q1 (n = 835)	Q2 (n = 833)	Q3 (n = 834)	Q4 (n = 832)	Q5 (n = 835)	*P*
Age (years)	57.18±8.32	57.48±8.38	56.34±8.51	53.99±8.65	51.92±8.55	<0.0001
Male	293 (35.09)	298 (35.77)	351 (42.09)	406 (48.8)	482 (57.72)	<0.0001
Education level						
Under high school	693 (83.9)	704 (85.64)	662 (80.15)	586 (71.29)	499 (60.12)	<0.0001
Graduated high school	104 (12.59)	89 (10.83)	129 (15.62)	178 (21.65)	238 (28.67)	.
Some college or higher	29 (3.51)	29 (3.53)	35 (4.24)	58 (7.06)	93 (11.2)	.
Monthly income (KRW), %						
< 1,000,000	536 (65.85)	540 (66.26)	489 (59.63)	410 (50.43)	318 (39.07)	<0.0001
1,000,000 ≤ 1,999,999	202 (24.82)	189 (23.19)	219 (26.71)	258 (31.73)	299 (36.73)	
≥ 2,000,000	76 (9.34)	86 (10.55)	112 (13.66)	145 (17.84)	197 (24.20)	
BMI (kg/m^2)	24.68±3.4	24.37±3.38	24.3±3.26	24.45±3.17	24.48±3.28	0.18
Current smoking	175 (21.34)	187 (22.72)	205 (24.88)	247 (30.05)	291 (35.4)	<0.0001
Alcohol intake (g/day)						
Non-intake	559 (69.61)	534 (65.84)	477 (59.48)	439 (54.33)	325 (41.4)	<0.0001
< 15.0 g/day	166 (20.67)	182 (22.44)	219 (27.31)	204 (25.25)	252 (32.1)	·
15.0–24.9g/day	27 (3.36)	33 (4.07)	38 (4.74)	65 (8.04)	63 (8.03)	·
≥ 25.0 g/day	51 (6.35)	62 (7.64)	68 (8.48)	100 (12.38)	145 (18.47)	·
Physical activity[1] (MET-hours/week)						
Q1 (< 25th)	174 (20.84)	159 (19.09)	155 (18.59)	183 (22)	196 (23.47)	0.10
Q2 (25–49th)	105 (12.57)	130 (15.61)	119 (14.27)	117 (14.06)	131 (15.69)	.
Q3 (50–74th)	158 (18.92)	159 (19.09)	172 (20.62)	170 (20.43)	173 (20.72)	.
Q4 (≥ 75th)	398 (47.66)	385 (46.22)	388 (46.52)	362 (43.51)	335 (40.12)	.
Parental history of CVD	27 (3.23)	22 (2.64)	24 (2.88)	24 (2.88)	33 (3.95)	0.58
History of DM	29 (3.51)	29 (3.53)	35 (4.24)	58 (7.06)	93 (11.2)	0.51
History of dyslipidemia	9 (1.08)	9 (1.08)	14 (1.68)	16 (1.92)	11 (1.32)	0.50
History of HTN	166 (19.88)	153 (18.37)	144 (17.27)	151 (18.15)	125 (14.97)	0.11
Number of metabolic diseases						
0	621 (74.37)	641 (76.95)	640 (76.74)	641 (77.04)	671 (80.36)	0.008
1	184 (22.04)	168 (20.17)	171 (20.5)	156 (18.75)	125 (14.97)	·
≥ 2	30 (3.59)	24 (2.88)	23 (2.76)	35 (4.21)	39 (4.67)	·

BMI, body mass index; MET, metabolic equivalent of task; KRW, Korean Won; CVD, cardiovascular disease; DM, diabetes mellitus; HTN, hypertension

Supplemental Table S4. Baseline characteristics of the subjects in the industrial region according to animal-based DP quintile score

**Characteristic**	Q1 (n= 836)	Q2 (n=837)	Q3 (n=837)	Q4 (n=836)	Q5 (n=837)	*P*
Age (years)	49.91±8.08	49.52±7.9	48.78±7.67	48±7.16	47.14±6.66	<0.0001
Male	338 (40.43)	390 (46.6)	438 (52.33)	441 (52.75)	550 (65.71)	<0.0001
Education level						
Under high school	347 (41.51)	338 (40.43)	288 (34.41)	279 (33.37)	217 (26.02)	<0.0001
Graduated high school	331 (39.59)	349 (41.75)	370 (44.21)	373 (44.62)	394 (47.24)	
Some college or higher	158 (18.9)	149 (17.82)	179 (21.39)	184 (22.01)	223 (26.74)	
Monthly income (KRW), %						
< 1,000,000	137 (16.57)	144 (17.33)	106 (12.74)	88 (10.56)	53 (6.34)	<0.0001
1,000,000 ≤ 1,999,999	291 (35.19)	264 (31.77)	259 (31.13)	238 (28.57)	209 (25.00)	
≥ 2,000,000	399 (48.25)	423 (50.90)	467 (56.13)	507 (60.86)	574 (68.66)	
BMI (kg/m^2)	24.71±3	24.55±3	24.68±2.84	24.69±2.95	24.95±2.97	0.10
Current smoking	138 (16.59)	165 (19.78)	197 (23.56)	228 (27.34)	269 (32.18)	<0.0001
Alcohol intake (g/day)						
Non-intake	508 (61.5)	438 (52.77)	406 (49.03)	355 (42.87)	280 (33.57)	<0.0001
< 15.0 g/day	228 (27.6)	265 (31.93)	246 (29.71)	255 (30.8)	241 (28.9)	
15.0–24.9g/day	38 (4.6)	51 (6.14)	79 (9.54)	79 (9.54)	94 (11.27)	
≥ 25.0 g/day	52 (6.3)	76 (9.16)	97 (11.72)	139 (16.79)	219 (26.26)	
Physical activity[1] (MET-hours/week)						
Q1 (< 25th)	239 (28.59)	210 (25.09)	198 (23.66)	188 (22.49)	183 (21.86)	0.03
Q2 (25–49th)	335 (40.07)	340 (40.62)	340 (40.62)	326 (39)	346 (41.34)	
Q3 (50–74th)	216 (25.84)	248 (29.63)	263 (31.42)	275 (32.89)	255 (30.47)	
Q4 (≥ 75th)	46 (5.5)	39 (4.66)	36 (4.3)	47 (5.62)	53 (6.33)	
Parental history of CVD	44 (5.26)	30 (3.58)	32 (3.82)	38 (4.55)	32 (3.82)	0.41
History of DM	41 (4.9)	46 (5.5)	54 (6.45)	38 (4.55)	43 (5.14)	0.47
History of dyslipidemia	34 (4.07)	28 (3.35)	20 (2.39)	31 (3.71)	24 (2.87)	0.32
History of HTN	103 (12.32)	101 (12.07)	98 (11.71)	82 (9.81)	88 (10.51)	0.42
Number of metabolic diseases						
0	684 (81.82)	687 (82.08)	683 (81.6)	709 (84.81)	696 (83.15)	0.39
1	129 (15.43)	126 (15.05)	137 (16.37)	106 (12.68)	127 (15.17)	
≥ 2	23 (2.75)	24 (2.87)	17 (2.03)	21 (2.51)	14 (1.67)	

BMI, body mass index; MET, metabolic equivalent of task; KRW, Korean Won; CVD, cardiovascular disease; DM, diabetes mellitus; HTN, hypertension

Supplemental Table S5. Sensitivity analysis of the association between an animal-based DP and incident CVD

Industrial region	Quintile of the animal-based DP score					*P* for trend
	Q1	Q2	Q3	Q4	Q5	
Subtype of CVD						
Coronary artery disease	ref	1.13 (0.57-2.25)	0.88 (0.43-1.81)	1.21 (0.6-2.42)	0.34 (0.12-0.96)	0.14
Stroke	ref	0.93 (0.51-1.71)	0.67 (0.35-1.3)	0.65 (0.33-1.29)	0.31 (0.13-0.73)	0.005
Myocardial infarction	ref	1.35 (0.45-4.09)	1.12 (0.36-3.5)	0.53 (0.13-2.14)	1.05 (0.32-3.37)	0.60
Subgroup						
Current smoker (n = 997)	ref	1.03 (0.44-2.41)	0.39 (0.14-1.09)	0.61 (0.25-1.5)	0.55 (0.23-1.34)	0.09
BMI ≥ 25.0 kg/m^2 (n = 1,837)	ref	0.76 (0.42-1.37)	0.59 (0.31-1.11)	0.83 (0.47-1.49)	0.38 (0.19-0.77)	0.02
< 60 years old (n = 3,666)	ref	1.11 (0.66-1.85)	0.77 (0.44-1.35)	0.92 (0.53-1.60)	0.48 (0.25-0.91)	0.02

DP, dietary pattern; CVD, cardiovascular disease; BMI, body mass index
Hazard ratios with 95% confidence intervals were obtained after adjustment for age, sex, educational level, smoking status, alcohol intake, quartile of physical activity, parental history of cardiovascular disease, body mass index, and number of metabolic diseases at baseline.
